# Quantitative in-vitro assessment of a novel robot-assisted system for cochlear implant electrode insertion

**DOI:** 10.1007/s11548-024-03276-y

**Published:** 2024-10-01

**Authors:** Philipp Aebischer, Lukas Anschuetz, Marco Caversaccio, Georgios Mantokoudis, Stefan Weder

**Affiliations:** 1 Hearing Research Laboratory, ARTORG Center for Biomedical Engineering Research, University of Bern, Bern, 3008 Switzerland; 2 Department for Otolaryngology, Head and Neck Surgery, Inselspital University Hospital Bern, 3010 Bern, Switzerland; 3 Department of Otorhinolaryngology, Head and Neck Surgery, Lausanne University Hospital (CHUV), University of Lausanne, 1011 Lausanne, Switzerland; 4 The Sense Innovation and Research Center, 1011 Lausanne and Sion, Switzerland

**Keywords:** Cochlear implant, Robot-assisted surgery, Hearing preservation, Soft surgery, Insertion force, Intracochlear pressure

## Abstract

**Purpose:**

As an increasing number of cochlear implant candidates exhibit residual inner ear function, hearing preservation strategies during implant insertion are gaining importance. Manual implantation is known to induce traumatic force and pressure peaks. In this study, we use a validated in-vitro model to comprehensively evaluate a novel surgical tool that addresses these challenges through motorized movement of a forceps.

**Methods:**

Using lateral wall electrodes, we examined two subgroups of insertions: 30 insertions were performed manually by experienced surgeons, and another 30 insertions were conducted with a robot-assisted system under the same surgeons’ supervision. We utilized a realistic, validated model of the temporal bone. This model accurately reproduces intracochlear frictional conditions and allows for the synchronous recording of forces on intracochlear structures, intracochlear pressure, and the position and deformation of the electrode array within the scala tympani.

**Results:**

We identified a significant reduction in force variation during robot-assisted insertions compared to the conventional procedure, with average values of 12 mN/s and 32 mN/s, respectively. Robotic assistance was also associated with a significant reduction of strong pressure peaks and a 17 dB reduction in intracochlear pressure levels. Furthermore, our study highlights that the release of the insertion tool represents a critical phase requiring surgical training.

**Conclusion:**

Robotic assistance demonstrated more consistent insertion speeds compared to manual techniques. Its use can significantly reduce factors associated with intracochlear trauma, highlighting its potential for improved hearing preservation. Finally, the system does not mitigate the impact of subsequent surgical steps like electrode cable routing and cochlear access sealing, pointing to areas in need of further research.

## Introduction

Cochlear implants represent a major advance in auditory prosthetics, enabling hearing in individuals with profound deafness. Recently, indications have expanded to patients with residual hearing, where atraumatic electrode placement is particularly important for optimal hearing outcomes. The delicate structures of the auditory organ challenge manual insertion techniques, limited by human kinematic abilities [1]. Studies highlight the risks of intracochlear damage from manual methods, underscoring the need for improved techniques [2–4]. In this context, robot-assisted systems could offer a solution.

The first robotic device for cochlear implant electrode placement was developed over a decade ago by Hussong et al. It utilized two independent actuators for perimodiolar array insertion [5]. Later work by Schurzig et al. added force measurement capabilities, though the design did not progress to clinical application [6, 7]. Subsequently, a simpler system for inserting straight electrodes, the iotaSOFT System (iotaMotion, Inc., Iowa City, USA), was introduced [3]. In addition, the RobOtol (Collin, Bagneux, France), an articulated arm designed for broader otologic applications, is used for inserting straight electrodes and for securing the straightener of perimodiolar electrodes [8]. Our team has developed a head-mounted system that features real-time force monitoring [9].

Most recently, the field has gained a new addition with OTODRIVE (CASCINATION AG, Bern, Switzerland), a novel device that enables the motorized forward movement with user-defined speed. This feature would enable the insertion of cochlear implant electrode arrays into the inner ear. In this study, we perform a comprehensive, quantitative evaluation of OTODRIVE using a validated in-vitro model of the temporal bone. Our evaluation compares established indicators of cochlear trauma, including insertion force and intracochlear pressure, between the conventional manual procedure and robotic assistance during all steps of the implantation.

## Methods

### Device description

**Fig. 1:**
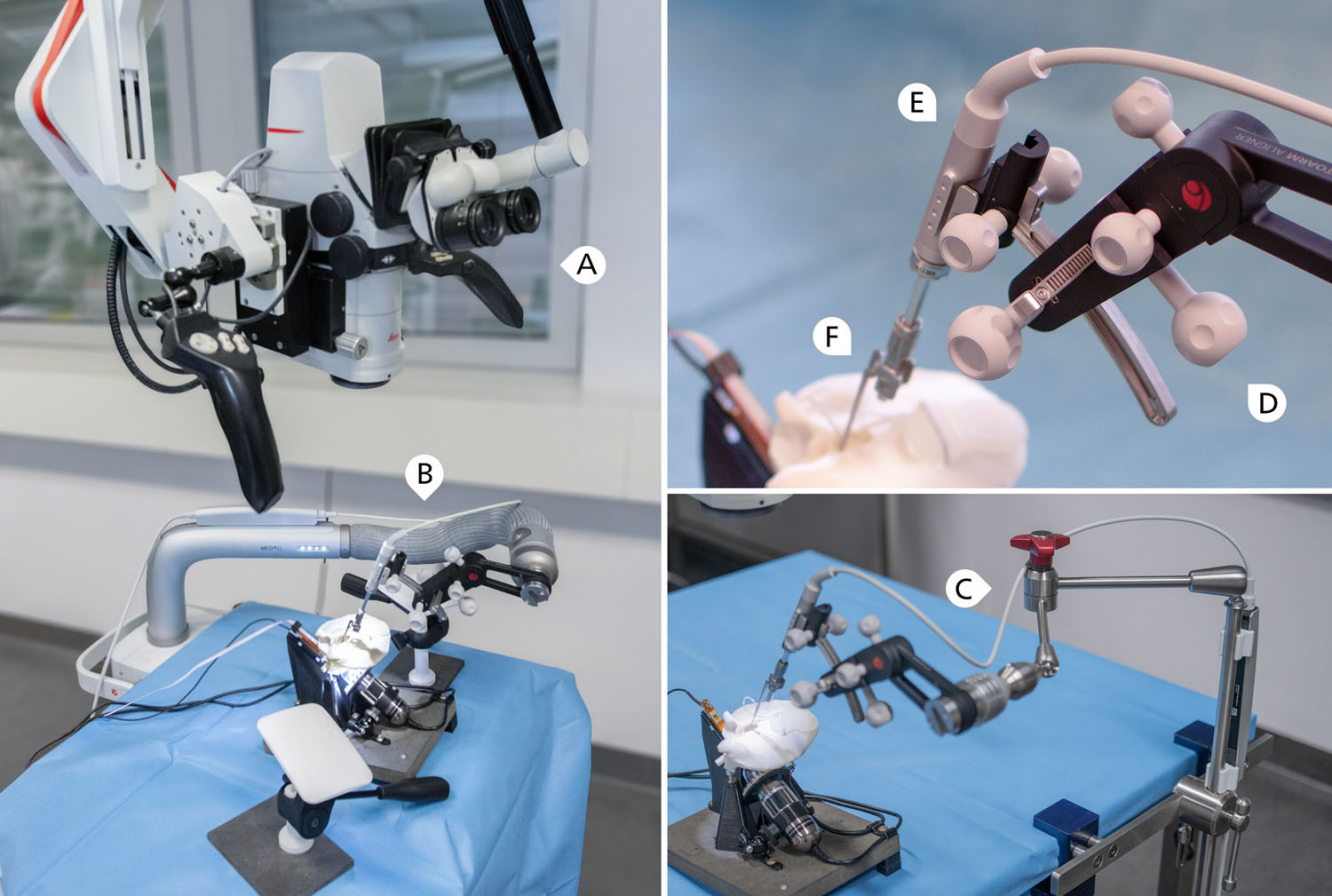
Left: Experimental setup with surgical microscope (**A**) and the robot-assisted insertion device mounted to a positioning arm (**B**). Lower right: Due to limited availability the positioning arm was replaced with a medical articulated arm (**C**) for 22 insertions. Hand rests that simulate patient extent are removed in this photograph for better visual overview. Upper right: Close-up of the assessed device, consisting of *OTOARM Aligner* (**D**) with the *OTODRIVE Handpiece* (**E**) mounted and *forceps OD* (**F**) partially extended into the temporal bone model Manual procedures were conducted in the same setup with the positioning arm and insertion device removed

The setup is comprised of four main parts which include (A) a positioning arm, (B) an alignment unit (*OTOARM Aligner*), (C) a linear actuator (*OTODRIVE Handpiece*) operated by a laptop computer and foot pedal and (D) a self-closing forceps (*Forceps OD*). Figure 1 shows the complete setup and a close up of the actuator end. Each component is described in detail below.

#### OTODRIVE

OTODRIVE handpiece features a metal shaft with an internal magnetic core that moves along the shaft, magnetically coupling with the forceps, allowing for a total travel of 40 mm. The OTODRIVE handpiece is connected to the OTODRIVE box, which serves as the control unit and offers Blutooth or Ethernet connection to a computer running the OTODRIVE software. The software allows adjustment of the feed rate in ten uniform steps from 0.1 mm/s to 1 mm/s and visualizes the current actuator position. It also permits setting a relative zero position, shown alongside the current absolute position. The OTODRIVE foot pedal, equipped with forward and backward switches, also connects to the OTODRIVE box by which the user activates the motion and its direction. The OTODRIVE handpiece includes an interface to be attached to the OTOARM Aligner.

#### Positioning arm

An electro-mechanical, table mounted positioning arm was used for a subset of the robot-assisted insertion experiments. The arm is attached to the contralateral side railing of the operating table. It reaches superiorly around the patient’s head, up to a position posterior to the surgical site. Activating a button on the distal end transitions the arm into a flexible configuration, allowing to reposition the acutator. After initial placement, the device position can be fine-tuned with OTOARM Aligner. It features manually operated knobs for adjustments across three linear axes: inferior to superior (10 mm range), anterior to posterior (10 mm range), and along the actuator axis (26 mm range). It allows adjustments along two rotational axes to determine the medio-lateral and basoapical insertion angles (20$^{\circ }$ range).

Due to limited availability of the electro-mechanical positioning arm for 22 robot-assisted insertions, a FISSO articulated arm (Baitella AG, Zürich, Switzerland) was used for positioning, and the OTOARM Aligner was attached using a customised adaptor.

### Study protocol

Sixty insertion experiments were conducted by three expert cochlear implant surgeons. Every surgeon executed 10 insertions using robotic assistance and 10 following the standard manual protocol. We alternated between manual and robot-assisted procedures for each insertion, to minimize the influence of electrode degradation.

The procedure for each experiment encompassed the entire implant placement process: electrode array insertion, positioning muscle tissue around the round window and into the facial recess, and coiling the electrode cable into the mastoid cavity. The steps were performed under a surgical microscope, with conventional surgical tools available. For robot-assisted insertions, additional steps included the initial placement and alignment of the device, fixation of the electrode, and instrument removal. For all manual tasks, including those performed after robotic assistance, surgeons were instructed to adhere to established soft surgery principles.

Before the expirements were started, each surgeon received an introduction to the robotic system’s operating components. Surgeons were permitted to familiarize with the tool within the in-vitro model until they felt proficient to proceed with the insertion experiments. For robot-assisted insertions, the feed-forward speed was set to 0.3 mm, which is a frequently used value [8, 10].

The measurements were conducted in two sessions per surgeon, with two new electrodes prepared for each session. Electrodes were replaced after six insertions. Three electrodes were replaced due to detected defects, for which three previously used electrodes were reutilized.

### Experimental setup

We used a validated in-vitro model within a realistic surgical environment. The setup includes a scala tympani model housed in a temporal bone model, derived from microtomography scans of human temporal bones. Access to the round window is through a standard mastoidectomy and posterior tympanotomy. The scala tympani model was fabricated by casting an ABS preform into clear epoxy resin and dissolving the preform post-curing, allowing for accurate reproduction of the 3D macro-anatomy without layer artifacts typical of 3D printing. Model and electrodes were coated with a hydrophilic polymer brush (graft copolymer with a poly(L-lysine) backbone and poly(ethylene glycol) side chains, PEG-g-PLL) to simulate realistic friction conditions. Details of the fabrication process and validation are discussed in a technical note [11].

To measure insertion forces, the temporal bone and scala tympani models are mechanically decoupled. The scala tympani is mounted to a load cell (KD78, ME Meßsysteme GmbH, Hennigsdorf, Germany), aligned with the long axis of the cochlea. The apex connects to a pressure sensor (MS5837-02BA, Measurement Specialities, Inc, Hampton, VA, USA). The model is oriented according to our standard clinical protocol, in the lateral position with the head hyperextended. The setup has been extensively described and utilized in previous studies [4, 9, 12].

Twelve MED-EL Flex28 electrode arrays (MED-EL GmbH, Innsbruck, Austria) were used, and connected to a dummy implant housing. Damaged arrays were replaced. A thin, flexible film (stretched Parafilm "M" laboratory film, Bemis Company, Inc, Neenah, WI, USA) with a hole (radius 0.25 mm) was placed at the entrance of the scala tympani model to mimic the round window membrane. This film was replace after each insertion.

The procedures were conducted under a surgical microscope (M525, Leica Microsystems GmbH, Wetzlar, Germany). Surgeons had access to specialized forceps (MED-EL SoftGrip Forceps), a surgical claw (MED-EL Surgical Claw Angled), a pick, and standard forceps. Porcine abdominal tissue was used for the placement of autologous tissue around the round window and within the posterior tympanotomy.

### Data processing

Raw data from load cell, pressure sensor, microscope image of the scala tympani and surgeon’s view was recorded with a custom python program. We post-processed data in python using the scipy scientific computing library [13]. In order to map the signals to the individual surgical steps, we extracted the corresponding timestamps from the video recording of the surgical microscope.

#### Force variation

Force variation was defined as the time derivative of the insertion force. Mean force variation was defined according to Nguyen et al. as the root mean square of the force variation [14].

#### Pressure peaks

Pressure peaks were identified using scipy’s *signal.find_peaks*. The strength was obtained by the value of the highpass-filtered signal, with a cutoff frequency set at 1 Hz. We classified strong pressure peaks as those exceeding 100 Pa, following the work of Greene et al. [15].

#### Angular and linear insertion depth

We manually annotated the positions of electrode contacts within the scala tympani model in the microscope recordings at 15-second intervals. These annotations were then tracked over time using the OpenCV library [16] and DaSiamRPN network for visual object tracking [17].

The angular insertion depth was defined as the azimuth coordinate of the uppermost electrode contact in the local cochlear coordinate system according to Verbist et al. [18]. For each video frame we obtained the electrode centerline by constructing a smooth spline approximation which connects the location of the round window and all intracochlear electrode contacts. The linear insertion depth was defined as the length of this electrode centerline.

#### Insertion speed

Insertion speed was defined as the time derivative of the linear insertion depth. For representing the inserton speed distribution, we normalized the data by distance. For this, we multiplied each bin count of the histogram of insertion speeds by its corresponding speed value.

#### Statistical analysis

The distribution of all measured variables was assessed using the Shapiro-Wilk test for normality. Measurands exhibiting normal distribution are presented as mean ± standard deviation. Measurands not conforming to a normal distribution are reported as median and interquartile range IQR.

### Forceps opening forces

Forceps opening forces were measured with a spring scale (SAUTER 281-752, Kern & SOHN GmbH, Balingen-Frommern, Germany).

## Results

### Positioning arm

Robot-assisted insertions were preformed with either an electro-mechanical positioning arm or a medical articulated arm. We observed no statistically significant differences in any measured parameters between the two mounting modalities: maximal forces ($p\,{=}\,0.67$), force variation ($p\,{=}\,0.80$), number ($p\,{=}\,0.93$) and strength ($p\,{=}\,0.55$) of pressure peaks, and insertion depth ($p\,{=}\,0.04$). Therefore, we perform the subsequent analysis on the two primary groups of robot-assisted and manual insertions.

### Device usage

The insertion of all electrode arrays was completed successfully, achieving a median depth of 558$^{\circ }$ (IQR 543$^{\circ }$–581$^{\circ }$), with no significant differences observed between manual and robot-assisted methods, as confirmed by the Kolmogorov-Smirnov test ($p\,{=}\,0.39$). Setting up the robot system went without any major difficulties. In two instances, minor realignment of the system was necessary during the insertion to prevent contact with the temporal bone. In another case, the forceps made contact with the posterior margin of the facial recess, hindering further progress until the issue was resolved by manual realingment. Tool removal was performed by handing over the electrode array either to the electrode forceps (9 cases), or stabilizing it against the margin of the facial recess with a surgical claw (21 cases), and subsequently opening and retracting the tool’s forceps. The tool removal was successful with a single exception where the electrode retracted to an angular depth of 360$^{\circ }$. In this case, the forceps were positioned too far into the facial recess. As a result, the surgical claw failed to hold the electrode in place when the forceps were opened.

We measured the force required to open the tool’s self-closing forceps at 9.7 N. For comparison, regular MED-EL Softgrip forceps require 1.2 N to close, while the self-closing Softgrip SR forceps require 2.1 N to open.

### Insertion speed and duration

**Fig. 2:**
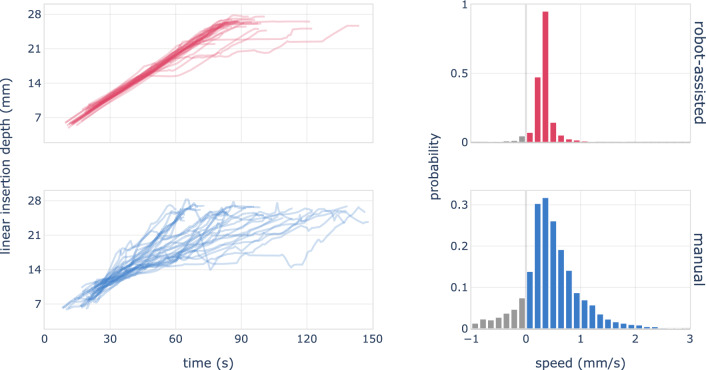
Linear insertion depth relative to time, and insertion speed distribution for robot-assisted (top) and manual (bottom) insertions. A consistent speed of 0.3 mm/s is evident in the robot-assisted data. Manual insertions display a broader range of speeds, including retractions and speeds above 0.5 mm/s, which are essentially absent under robotic assistance

We observed durations of the insertion of 98 s${\pm }$35 s and 103 s${\pm }$19 s for manual and robot-assisted insertions respectively. The durations are not different between the method of insertion ($p\,{=}\,0.50$, two-sample t-test), however the variance of manual durations is significantly wider (Bartlett’s test, $p\,{<}\,0.01$). A diagram showing the linear insertion depth of each insertion as a function of time is shown Fig. 2. The corresponding distribution of speed values is provided in the right-hand column of the same figure. Due to the implant fixation in robot-assisted insertions, there is only a negligible amount of backward movement of the electrode within the cochlea, which can occur due to manual contact with the insertion system or the extracochlear portion of the implant. In contrast, during manual insertion, we observed regular backward movement of the implant, which is related to the unevenness of manual movement [1].

### Insertion forces

**Fig. 3:**
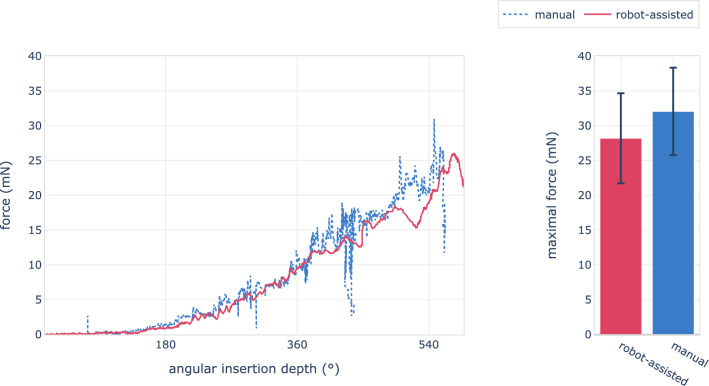
Left: Insertion force relative to angular insertion depth for a typical robot-assisted (red) and manual (blue) procedure. The robotic assistance provides a more even application of force. A characteristic pattern observed in manual insertions includes the cyclic release and reapplication of force without altering the implant’s position, as seen at 430$^{\circ }$. Beyond these variations, both methods exhibit similar force progression, determined primarily by the cochlea’s geometry. Right: Mean and standard deviation of maximum forces recorded across all trials, categorized by the insertion method

**Fig. 4:**
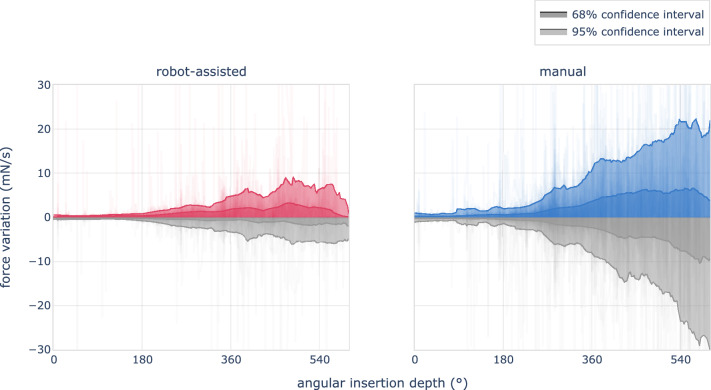
Force variation of robot-assisted (left) and manual (right) insertions versus angular insertion depth. Positive values correspond to an increased load on intracochlear structures. Negative force values reflect a decrease in applied force and are presented in gray. Robot-assisted insertions apply substantial lower force variation on intracochlear structures, with mean values of 12 mN/s, as compared to 33 mN/s for manual insertions

Maximal insertion forces amounted to 32 mN ${\pm }$ 6 mN and 28 mN ${\pm }$ 6 mN for manual and robot-assisted insertions respectively. This difference falls short of significance (Kolmogorov-Smirnov test, $p\,{=}\,0.016$). Figure 3 shows the force of a typical manual and robot-assisted insertion, together with the distribution of maximal insertion forces of all procedures. Contrary to maximal force, force variation differs significantly with values of 33 mN/s ${\pm }$ 26 mN/s and 12 mN/s ${\pm }$ 8 mN/s respectively ($p\,{<}\,0.001$). Force variation versus insertion depth are shown in Fig. 4.

**Fig. 5:**
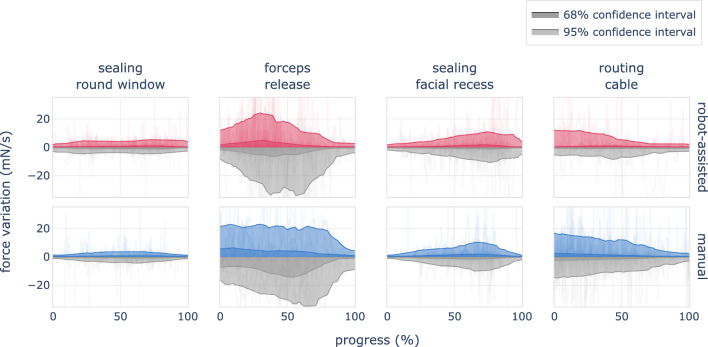
Force variation of surgical steps after the insertion. The use of robotic assistance introduces the tool’s removal as an additional step, which is included in the second column. During this phase, we observed significant force variation, indicating that surgeons must practice this step diligently and proceed with caution. No significant differences were observed between manual and robot-assisted procedures in any of the four post-insertion steps

Substantial force variation continued during post-insertion steps, which included placement of tissue around the round window and in the facial recess, releasing of the forceps and routing of the electrode lead cable into the mastoid cavity. The force variation of these four post-insertion steps is shown in Fig. 5.

### Intracochlear pressure

**Fig. 6:**
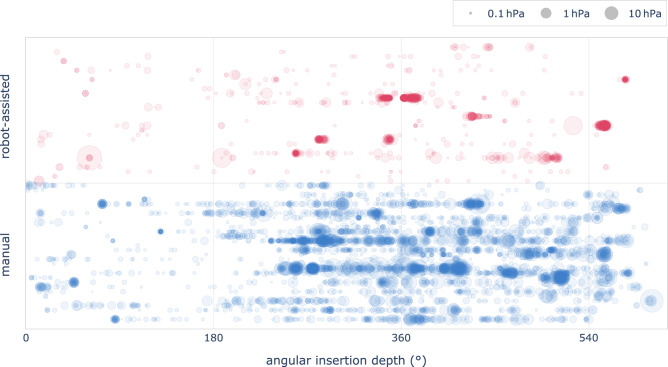
Pressure peaks during the insertion of the electrode array. Each horizontal line corresponds to a single insertion, disk size relates logarithmically to pressure strength

Intracochlear pressure levels were reduced by 17 dB with robotic assistance when compared to manual insertions. We observed a reduction in number and strength of pressure peaks. Pressure peaks of all insertions are shown in Fig. 6. We observed no strong pressure peaks (above 100 Pa) within the first three quartiles of robot-assisted insertions, but a median count of 5 (IQR 0−8) for manual insertions. This difference is statistically highly significant (Kolmogorov-Smirnov test, $p\,{<}\,0.001$). A substantial portion of pressure peaks occured after insertion of the electrodes, resulting in a median count of 8 (IQR 2−22) strong peaks for the complete robot-assisted and 10 (IQR 0−28) for the complete manual procedures.

## Discussion

In this study, we evaluate a novel system designed for the robot-assisted placement of cochlear implant electrode arrays. We compare key metrics related to intracochlear stress between manual and robot-assisted insertions using a realistic in-vitro temporal bone model. A total of 60 insertions were performed by three expert cochlear implant surgeons.

### Reduction of intracochlear stress

Robotic assistance enables a substantially smoother movement of the electrode array, as vividly illustrated in Fig. 2. A steadier electrode motion results in reduced variation in forces exerted on the cochlea. The impact is captured in Fig. 4, and our data reveal a nearly threefold decrease in mean force variation, from 33 mN/s down to 12 mN/s.

The main contributors to insertion forces are the friction resistance and elastic restoring force. These two fundamental force components exist irrespective of the insertion method. Consequently, the observed insertion forces are similar between manual and robot-assisted methods, with forces exponentially increasing with the insertion depth. However, the additional force variation observed in manual insertions is superimposed on these underlying components, leading to higher peak forces. Indeed, we observed a trend towards lower peak forces with robotic assistance, although this difference did not reach statistical significance.

The motorized movement reduces variation in perilymph displacement, which positively affects intracochlear pressure. Our findings include a notable reduction in intracochlear pressure levels by 17 dB. We have defined significant pressure peaks as spikes exceeding 100 Pa which corresponds to an approximate sound pressure level of 134 dB within the ear canal [15]. Such intense pressure peaks are capable of causing long-term damage to intracochlear mechano-sensory and neural structures [19, 20]. Robotic assistance significantly mitigates these occurrences, as evidenced by an average of only 0.7 peaks per insertion, in stark contrast to an average 12.4 peaks observed during manual procedures. This translates to a reduction of strong pressure peaks by a factor of 17.

During robotic insertions, strong pressure peaks occurred in 10 instances. From retrospective analysis, we can attribute half to vibrations from surgeon activities, including accidental arm contact (3 incidents) and adjustments to the aligner (2 incidents). One instance resulted from manual electrode manipulation, with the remaining unlinked to any specific interaction. This underscores the importance of minimizing external vibrations by omitting contact with the implant and robotic system.

### Post-insertion management

The procedure introduces an additional step to the surgical sequence, which is the handover of the electrode after its insertion. Our measurements show that this step is a significant contributor to force variation and pressure peaks (see Fig. 5). Its impact is comparable to the release of the implant in the manual procedure. We therefore recommend comprehensive training of the handover.

Releasing the tool is a more complex task than releasing the implant in the manual procedure, as it requires to push open the tool’s self-closing forceps and removing the device with one hand while stabilizing the electrode with the other hand. We measured the opening force to be substantially higher than the force required to close a regular cochlear implant forceps or to open the self-closing version. This increased force requirement may reduce the precision of the surgeon’s movements.

Current robotic systems automate electrode insertion but don’t address post-insertion steps, which are crucial for atraumatic implantation as highlighted by recent studies [4, 21]. Our data show that manual implantation and subsequent implant management steps contribute equally to the number of strong pressure peaks. Although robot-assisted insertion significantly reduces pressure peaks, manual post-insertion steps are still necessary. Consequently, when considering the entire procedure, robotic assistance reduces the total number of strong pressure spikes by a factor of two.

### Instrument handling

Robotic assistance alters the surgical workflow which offers both challenges and opportunities. Manual cochlear implant forceps facilitate visual access with their angled tips, and surgeons can adjust the position of the forceps for an optimal view of the structures. Conversely, robotic systems secure the electrode in a fixed position and allow adjustment of the viewing angle of the surgical microscope while maintaining the electrode stable.

In conventional cochlear implantation, surgeons use tactile feedback for detecting resistance that might damage cochlear structures. However, such feedback is lacking in current robotic systems. Increased forces may be detected through extracochlear electrode buckling, but this method remains qualitative. The correlation between buckling and insertion forces is not known and may be influenced by factors such as tool alignment and grasping position.

We recorded an instance where the forceps contacted the facial recess, preventing further movement. Due to the slow movement, this is not immediately evident. Since the internal magnet coupling to the forceps continues to advance, a forward force arises, and when the alignment is corrected, the forceps will abruptly realign with the actuator. In the documented case, this resulted in a sudden 1.8 mm increase in insertion depth and a 14 mN force spike. This highlights the necessity for precise initial alignment and continuous monitoring of the tool’s position. Should contact occur, the operator must retract the tool until the obstruction is resolved before attempting realignment.

### Integration with other tools

Robot-assisted systems can be used together with other currently employed monitoring tools. Notably, the increased stability of the electrode position is expected to improve signal quality of electrophysiological measurements such as electrocochleography [22]. Ultimately, electrophysiological monitoring may support robot-assisted insertion, by providing information about the exact intracochlear placement [23, 24] and health of cochlear structures [25, 26].

In addition, we see potential for adding force measurement capability to robot-assisted systems. Such tools can improve force sensitivity beyond human perception and help detect traumatic events [9].

Finally, our team previously showed that intracochlear electrode redirection allows to reduce insertion forces, by reducing the contact surface and therefore frictional resistance between the electrode and the scala tympani [12]. Such tools might be used in conjunction with robot-assisted devices to further reduce insertion forces.

### Limitations

This study utilized an in-vitro model of the temporal bone, which bases on the established relationship between insertion forces, intracochlear pressure peaks, and intracochlear trauma [2, 27–30]. The in-vitro setting offers controlled and repeatable conditions; however, it also implies that the experiments were conducted on a single specimen with standard anatomy. Future studies need to explore the effectiveness of robot-assisted cochlear implantation across different anatomical variations and in cases of malformations.

Furthermore, we selected an insertion speed of 0.3 mm/s, consistent with other work [8, 10] and resulting in comparable insertion duration compared to the manual approach. The evaluated system allows insertion speeds down to 0.1 mm/s, and it has been shown that slower speeds and decreasing speed profiles allow to further reduce insertion forces [31, 32]

Device set up and alignment prolongs the duration of the surgery. Our study design focussed on the effects of electrode array insertion on the cochlea and is not suited for determining the setup time. The repeated alignment in a single anatomy could lead to an unrealistic learning effect, and the lack of a sterile drape could facilitate manipulation of the aligner, potentially underestimating the required setup time. We plan to perform a detailed analysis of the setup time and other usability-related parameters based on questionnaire feedback and adverse events in future research. Our initial clinical experience suggests that the system can be set up and aligned in about five minutes

## Conclusion

In this study, we conducted a comprehensive, quantitative assessment of a new system designed for the robot-assisted insertion of cochlear implant electrode arrays, comparing its performance with the conventional manual procedure.

The tool offers substantial improvements, particularly in achieving more consistent insertion speeds and significantly reducing factors associated with intracochlear trauma, including force variation and intracochlear pressure peaks. Notable findings include a 17dB decrease in intracochlear pressure and a reduction of strong, potentially traumatic, pressure peaks by a factor of 17. Furthermore, robotic assistance substantially reduces force variation during the insertion, from 33 mN/s to 12 mN/s. These results suggest potential for enhanced hearing preservation in cochlear implantation.

Finally, we have identified the handover of the electrode cable after the insertion as a critical point where significant forces and pressure peaks can act on the cochlea. Furthermore, current robot-assisted tools do not encompass solutions for subsequent surgical steps equally important to structural preservation, such as the routing of the electrode cable and the sealing of the round window and middle ear access. These results emphasize the need for comprehensive surgical training, even with the adoption of robot-assisted cochlear implantation techniques and point to areas in need of further innovation.
